# A Review of the “Omics” Approach to Biomarkers of Oxidative Stress in *Oryza sativa*

**DOI:** 10.3390/ijms14047515

**Published:** 2013-04-08

**Authors:** Nyuk Ling Ma, Zaidah Rahmat, Su Shiung Lam

**Affiliations:** 1 Department of Biology, Faculty of Science and Technology, University Malaysia Terengganu, 21030 Kuala Terengganu, Terengganu, Malaysia; 2 Department of Biotechnology and Medical Engineering, Faculty of Biosciences and Medical Engineering, University Technology Malaysia, 81310 Johor Bahru, Johor, Malaysia; E-Mail: zaidah@fbb.utm.my; 3 Department of Engineering Science, Faculty of Science and Technology, University Malaysia Terengganu, 21030 Kuala Terengganu, Terengganu, Malaysia; E-Mail: lam@umt.edu.my

**Keywords:** biomarker, stress signaling, “omic” study, metabolomic, proteomic, transcriptomic

## Abstract

Physiological and ecological constraints that cause the slow growth and depleted production of crops have raised a major concern in the agriculture industry as they represent a possible threat of short food supply in the future. The key feature that regulates the stress signaling pathway is always related to the reactive oxygen species (ROS). The accumulation of ROS in plant cells would leave traces of biomarkers at the genome, proteome, and metabolome levels, which could be identified with the recent technological breakthrough coupled with improved performance of bioinformatics. This review highlights the recent breakthrough in molecular strategies (comprising transcriptomics, proteomics, and metabolomics) in identifying oxidative stress biomarkers and the arising opportunities and obstacles observed in research on biomarkers in rice. The major issue in incorporating bioinformatics to validate the biomarkers from different omic platforms for the use of rice-breeding programs is also discussed. The development of powerful techniques for identification of oxidative stress-related biomarkers and the integration of data from different disciplines shed light on the oxidative response pathways in plants.

## 1. Introduction

Rice is still a staple food in many countries [1]. An increase in the human population places a great deal of pressure on the agriculture sector [2,3], in which the production of the rice can no longer fulfill worldwide demands [4]. In addition, the full yield potential of rice cannot be achieved due to environmental constraints such as temperature, drought, salinity, osmotic stress, *etc.* The relationship between rice and its environmental changes has been extensively studied in order to tackle the most important agronomical traits for sustainable rice production. It is believed that molecular studies that integrate pillars of proteins, metabolites, and transcripts play an indispensable role in the era of functional genomics. Interestingly, a key feature that stands out over all the general perspectives on plant’s response to stress is the enhanced production of reactive oxygen species (ROS), especially the production of hydrogen peroxide (H_2_O_2_) within sub-cellular compartments of the plant cell [5]. H_2_O_2_ is a more important secondary messenger than other ROS due to its relatively longer half-life and the fact that it is a small molecule, thus enabled to be transported through different cell compartments [6].

The production of H_2_O_2_ in plant functions as a double-edged sword: the basal level of H_2_O_2_ is important to maintain routine metabolic activities [7], whilst overproduction of H_2_O_2_ could lead to oxidative damage and kill the cells [8]. H_2_O_2_ involves in a number of signaling cascades in plants under stress conditions [9], acting as a defense signaling response to pathogen elicitors [10,11], triggering mitogen-activated protein (MAP) kinase and stomatal response [12,13], and initiating programmed cell death [14,15]. In extreme stress conditions, excess ROS accumulated in plant can lead to uncontrolled oxidative stress on DNA [16]. ROS can cause a DNA strand to break by modification either at deoxyribose sugar or at purine and pyrimidine bases [17]. The damage of DNA leads to the reduction of protein synthesis, and this contributes to the destruction of cell membrane and damage to photosynthetic proteins [18]. ROS can oxidize cysteine, tyrosine, tryptophan, histamine, and methionine [19]. Most of the protein oxidation reactions are irreversible [20] and only a few of which involve sulfur-containing amino acids are reversible [21]. The rapid and reversible modification reactions of a protein appears to be an ideal candidate for a signaling event, e.g., cysteine reacts only with H_2_O_2_[19] that gives rise to different modified products and results in activation of different sets of genes [22]; the oxidation of thiol residues of tyrosine dephosphorylation has been proved to mediate the signaling event in order to avoid programmed cell death caused by severe stress [23]. The sulfonic acid oxidation and carbonylation appear to be the most commonly occurring irreversible oxidative protein modification [24]. The OH• radical reacts with arginine, histamine, lysine, proline, threonine, and tryptophan and produces nearly 4 nmol/mg of carbonyl groups in stressed plant [25]. A higher concentration of carbonyl groups has been reported in senescence leaf, recording a concentration of approximately 600–700 nmol/mg in pea [26]. ROS has also been reported to have caused massive damage to the lipid group by reacting with the olefinic group and removing the allylic hydrogen atom in order to produce lipid peroxy radical. This leads to the breakdown of PUFA and in turn causes secondary damage to plant cells [19]. The adventitious peroxidation of neighboring unsaturated lipids by these radicals weakens the membrane fluidity, hence increasing the leakiness of membrane and leading to secondary damage to membrane proteins [19]. Moderate oxidative stress usually stops the cell cycle, but severe damage might trigger death by apoptosis, necrosis, or both [7]. Even though abiotic stresses have been shown to cause reduction in rice yield [27], the direct effects of ROS that lead to this depletion have not yet been reported. This is because the baseline level of H_2_O_2_ production often varies in different plant species and the complexity changes that occur under stress conditions have made it difficult to draw any conclusions [28]. Nevertheless, H_2_O_2_ has been reported to react with plant cells and it would leave traces of biomarkers at the genome, proteome and metabolome levels. In research studies, biomarkers can be employed to reflect the environmental pro-oxidant exposures and the antioxidants levels that serve as a surrogate measurement of the cellular status under certain stress condition. To date, the progress in the understanding of the oxidative stress mechanism in plant (particularly in rice) has been slow and frustrating due to the complex and heterogeneous nature of the oxidative molecule function in plant cells. The limited information available on oxidative molecule function has restricted the progress towards the improvement of crop production. Therefore, a close investigation into biomarkers for oxidative stress response could give a potential clue on how plant responses toward stress also provide early detection of ROS. In addition, stress biomarkers could reveal a suitable genes candidate for rice plant improvement. This review highlights the difficulties associated with identification of stress biomarkers, and the recent progress in “omics” technology towards the identification of biomarker in rice.

## 2. Cellular Sources and Regulation of ROS

**Figure 1:**
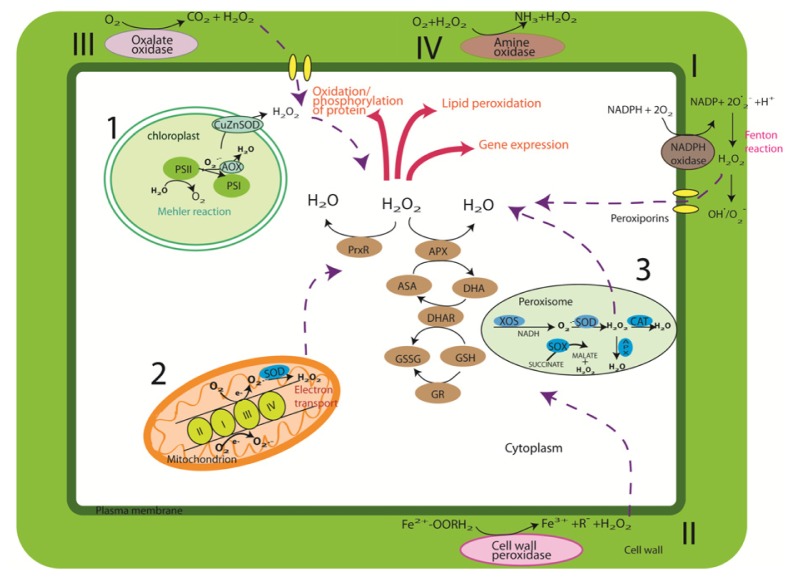
Biosynthesis of reactive oxygen species (ROS) and its regulation pathways in plant cells. The production and detoxification of ROS by various enzymatic pathways are shown. (1) The Fenton–Harber–Weiss cycle detoxifies O_2_^•−^ and H_2_O_2_ and alternative oxidase (AOX) reduces the production rate of O_2_^•−^ in thylakoids. In some plants, iron superoxide dismutase (FeSOD) might replace CuZnSOD in the chloroplast. H_2_O_2_ that escapes this cycle undergoes detoxification by SOD and the stromal ascorbate–glutathione cycle. Peroxiredoxin (PrxR) and glutathione peroxidase (GPX) are also involved in H_2_O_2_ removal in the stomata. (2) In mitochondria, SOD and other components of the ascorbate–glutathione cycle are induced following the production of H_2_O_2_. (3) In peroxisomes, ROS are scavenged by SOD, catalase (CAT), and ascorbate peroxidase (APX). The over-production of H_2_O_2_ is also generated and accumulated via (I) Plasma membrane-localized NADPH oxidase, (II) cell wall peroxidases, (III) germin-like oxalate oxidases, and (IV) amine oxidases. The NADPH-dependent oxidase system catalyzes the production of superoxide by reducing one oxygen electron using NADPH as a donor. The superoxide that is generated by this enzyme then converts to H_2_O_2_. Although the pathways in the different compartments are mostly separated from each other, H_2_O_2_ can easily diffuse through membranes, and antioxidants such as glutathione and ascorbic acid (reduced or oxidized) can be transported between the different compartments.

In the plant system, the majority of the ROS is generated from energy transfer during photosynthesis. When a plant is exposed to light, H_2_O_2_ is produced via the Mehler reaction in chloroplasts (1) and electron transport in peroxisomes (3) (Figure 1). Photorespiration in mitochondria (2) appears to be the main ROS producer for plants that were kept in darkness [29,30]. It was estimated that every 1%–5% of the O_2_ consumed contributed to ROS production [31]. ^1^O_2_ is a natural byproduct of photosynthesis in photosystem II (PSII), even under low light conditions. The photosynthetic electron transfer chain (ETC) is responsible for the production of H_2_O_2_. ETC components include a number of enzymes on the reducing side of photosystem I (PSI), namely: Fe–S center, reduced thioredoxin (TRX), and ferredoxin. Later studies have also suggested that the acceptor side of the electron transfer chain (ETC) in PSII provides electron leakage that changes O_2_ to O_2_^•−^ (Figure 1). H_2_O_2_ is then produced either through dismutation of O_2_^•−^ by superoxide dismutase enzymes (SOD) or through the reduction of superoxide using reductants such as ascorbate (ASA), thiols, and ferredoxins [32]. The reduction of H_2_O_2_ to H_2_O is achieved by oxidation of ASA to monodehydroascorbate radical (MDHA), which is catalyzed by ascorbate peroxidase (APX). The MDHA is subsequently reduced back to ASA by either ferredoxin reduction or NAD(P)H catalyzed chloroplastic MDHAR [33]. In peroxisomes, the main event occurred is photorespiration whereby O_2_^•−^ radicals are produced as a consequence of their normal metabolism. MDHAR mediates the formation of O_2_^•−^ in the peroxisome ETC, which is comprised of flavoprotein NADH and cytochrome *b*[34]. The main process that generates H_2_O_2_ in peroxisomes is via the enzymatic reaction of flavin oxidases [34,35]. The photorespiratory pathway appears to be a faster process to generate H_2_O_2_ compared to the Mehler reaction and electron transport in mitochondria [29]. Plant mitochondria act as an energy factory that contributes to the major production site of ROS [36]. The production of O_2_^•−^ occurs mainly at two sites of the electron transport chain: NAD(P)H dehydrogenases (Complex I) and the cytochrome *bc*1 complex (Complex III) [31]. The mitochondria component in plants differs from that in animals with specific ETC components that function in photorespiration. The ETC harbors electrons with sufficient free energy in order to directly reduce O_2_ in aerobic respiration. The superoxide anion then forms H_2_O_2_ via the reaction of mitochondrion-specific manganese SOD (Mn-SOD) [31]. The amount of H_2_O_2_ produced in plant mitochondria is less than that produced in chloroplasts or peroxisomes when the plant is exposed to light [29], while it appears to be a major ROS production site in the dark [37]. H_2_O_2_ reacts with reduced Fe^2+^ and Cu (produced from Fenton/Haber–Weiss reaction) to form reactive HO• that can penetrate cell membranes and travel in the cell [37].

Apart from the normal routes discussed above, ROS is also generated and accumulated via plasma membrane localized NADPH oxidase (I) and cell wall peroxidase (III) (Figure 1) [38]. The NADPH-dependent oxidase system catalyzes the production of superoxide by reducing one oxygen electron using NADPH as a donor. The superoxide that is generated by this enzyme then converts to H_2_O_2_. Biotic stress also increases H_2_O_2_ generation via the reaction of pH-dependent cell wall peroxidases (II), germin-like oxalate oxidases (III), and amine oxidases (IV) (Figure 1) [10]. Upon exposure to biotic stress, alkalinization of apoplast-activated cell wall peroxidase and the production of H_2_O_2_ is elicited [10].

## 3. Genomics of Rice

**Figure 2:**
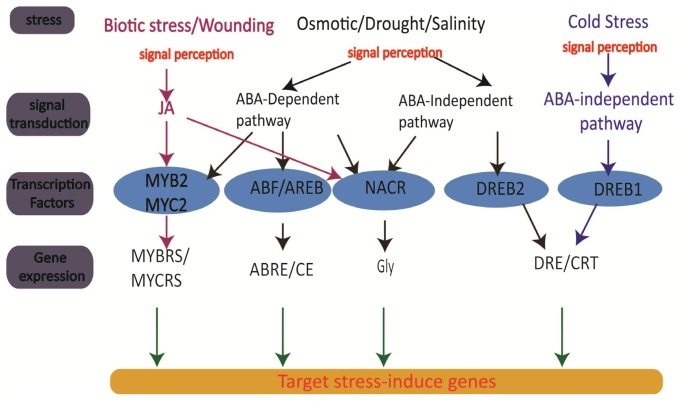
Transcription regulatory and gene expression networks of abiotic stress signals.

The initial strategy to improve crop production involves transformation of useful antioxidant genes into crop in an effort to produce stress tolerance plants (Table 1). However, it was found that common antioxidants are not suitable for all types of stress and hence stress-specific traits are needed to effectively target the different stress signaling pathways. Global analysis of transcription levels in rice plants under different abiotic stresses and in different organs have been intensively studied; a few examples include high salinity [39], drought [40], high temperature [41], cold and H_2_O_2_[42], and light [43]. However, in natural conditions, rice is exposed to various stresses simultaneously and, therefore, the responses toward stress adaptation are expected to be different compared to those exposed to single stress condition. Rabbani *et al.*[44] has proved the existence of significant crosstalk between stress-signaling pathways in their research on gene transcription integration under the combined treatment of cold, drought, salinity, and abscisic acid. Since then, numerous stress-induced genes have been identified and their functions are illustrated in Figure 2. The complex stress regulatory system is controlled by ABA divergent patterns. Dehydration-responsive element binding protein (DREB2) and NAC are examples of ABA-independent TFs [45,46], whereas the ABA-responsive element (ABRE) binding protein factor (ABF) functions in ABA-dependent gene expression [47]. In rice, five DREB cDNAs were identified, namely: Os*DREB*1A, Os*DREB*1B, Os*DREB*1C, Os*DREB*1D, and Os*DREB*2A [48]; Os*DREB*1A and Os*DREB*1B were found to be induced by cold stress while Os*DREB*2A was found to have the ability to regulate salt and drought stress [48]. Overexpression of Os*DREB*1A in rice could induce expression of DRE/CRT genes. It has been reported that the Os*DREB*1A overexpression lines show phenotypes similar to At*DREB*1A (*Arabidopsis*) overexpression line, but with improved stress tolerance [48].

There are 75 putative *NAC* genes in rice and these genes are mainly involved in regulating plant development and stress tolerance [60]. Salt and drought stress were found to induce *ERD1* expression via the ABA-independent pathway and they were recognized as *NACR* transcription factors [45]; this has been confirmed by Hu *et al.*[61], who have shown drought and salinity tolerance when the *NAC* genes were overexpressed. In addition to drought and salinity stress, Ohnishi *et al.*[62] have reported that Os*NAC*6, a member of *NAC* family, was induced under salinity, drought, cold, and ABA. Using the salt tolerant *indica* variety Nona Bokra *versus* to salt sensitive *japonica* variety Koshihikari, *Skc1*, loci for shoot K^+^ content that it involves in K^+^/Na^+^ homeostasis under salt stress, was demonstrated to confer salt tolerance at the seedling stage [63].

Three MYB transcription factors, *i.e.*, MYBS1, MYBS2, and MYBS3, each with a single DNA-binding domain (1R MYB), were identified in rice and they were found to have bound specifically to the TATA box (TATCCA) in the sugar response complex (SRC) of the a-amylase gene (aAmy3) promoter [64]. The MYBS3 in rice is activated by cold stress [65], abscisic acid (ABA), CdCl_2_, and NaCl [66]. In addition to these major pathways, MYB/MYC regulons have been reported to be involved in gene expression in wounding and biotic stress.

It has been reported in a study performed on *Arabidopsis*[67] that the transcription factors that are induced under oxidative stress show many similarities to that induced under ozone stress in rice plant [68], in which WRK, ERF (subfamily of DREB TF), NAM (NAC TF family), and MYB were identified. However, the transcription factors’ responses towards oxidative stress vary with different types of ROS. ^1^O_2_ represents the largest fraction of ROS-related genes while H_2_O_2_ and O_2_^•−^ responsive transcripts are mainly repressed [67]. To date, the ROS sensors and the signaling components that are responsible for this selectivity and specificity within the cell remains a major challenge. Using quantitative trait loci (QTL) analysis, *Sub1* locus that confers to submergence tolerance has been identified [69]. Three genes cluster, *i.e.*, *Sub1A*, *Sub1B*, and *Sub1C*, that encode putative ethylene response factors were observed and only *Sub1A* is variably present in the *Sub1* region. The two alleles that control the submergence ability, *i.e.*, *Sub1A-1* and *Sub1A-2*, were identified. Overexpression of *Sub1A-1* in submergence intolerance variety has demonstrated an enhanced tolerance to the plants.

The key research on genetic expression profile during oxidative stress in rice cultivar has been performed by using Affymetrix GeneChip Analysis [70]. A group of marker genes such as glutathione *S*-transferases (GSTs), P450, plant defense genes, and chalcone synthase (CHS) have been identified to play a role in oxidative stress mechanism.

## 4. Rice Proteomics

The analysis of a protein is the most direct approach to define the function of its associated genes. However, it must be mentioned that the genome and proteome of an organism do not always correspond to each other on a one-to-one basis [71,72]. Therefore, the analysis performed at the proteome and metabolome levels are equally important as the genomic study. The function of stress-induced proteins has been expanded by proteomic analysis in different tissues and organelles of rice plant, such as embryos [73], mitochondria [74], roots [75,76], leaves [77], anthers [78] and cell-suspension cultures [79]. A system for direct identification of proteins by differential display following 2-DE has been developed and the structure of the proteins can be identified either by comparison with the Rice Proteome Database (http://gene64.dna.affrc.go.jp/RPD/main.html), or by MS and Edman sequencing.

The major finding reported for protein markers of plant stress in rice, especially those that are sensitive to different stresses, are listed in Table 2. In addition to the antioxidants and redox group proteins that are expected to be identified in stressed plant, another protein group that functions in energy metabolism stands out as it seems to be activated almost in every stress. According to Cai and Tu [80], the proper regulation of the TCA cycle is very important in order to ensure a balance supply of energy to the building blocks of a cell. Therefore, the breaking of the normal energy metabolism is expected to interrupt the development of the cell, and the plant would have to undergo energy reserved state for survival. It was found that the recent proteomic studies in rice have so far focused on the identification of individual polypeptides based on their abundance when subjected to the treatment of different stresses (Table 2). The data of these complex physiological responses were found to vary over the time and stress degree, thus making the data comparison and the proteome integration analyses difficult to be performed between stress treatments. Hereafter, post-translational modification (PTM) such as phosphorylation and thiol modification caused by stress factors could be an alternative study to investigate stress signaling functions. Several strategies have been developed to identify PTM in plants. In particular, the development of 2D-PAGE technology coupled with the application of 5′-iodoacetamidofluorescein (5′IAF) [81,82] or MS such as in 2D-fluorescense difference gel electrophoresis (DIGE) [83] enables the identification of stress-related oxidized and reduced proteins. Many gel-free proteomic systems have also been developed for differential proteome analysis. Examples are multidimensional protein identification methods (MudPIT) that effectively identify individual components of proteins or peptides by avoiding band broadening for chromatographic detection [84], isotope-coded affinity tags (ICAT) [85], and isobaric tags for relative and absolute quantification (iTRAQ). These techniques are considered a targeted method to identify modification in proteins by means of the mass differences between treated and untreated proteomes. Nevertheless, limited studies have been performed on PTMs in plants due to: (1) the difficulties in applying the technique to plants, owing to the need of a large amount of protein for a reaction; (2) the changes of PTMs that often occur at different stages of growth and development, thus making it hard to detect; and, (3) the changing of proteins, which is detected as mass changes, requires complicated processing to transform these data into meaningful information.

## 5. Rice Metabolomics

Metabolites are the end products of cellular process and they reflect the response of biological systems to environmental changes [97]. The current trend in metabolomic studies is to define the cellular status at a particular time point by quantification of the total metabolites in the cellular system [98]. These techniques complement other techniques such as transcriptomics and proteomics and depict precise pictures of the whole cellular process. A range of analytical technology is available for plant metabolome study [99], including the use of high throughput approaches such as Fourier transform infrared (FT-IR) [100], ultra high-resolution Fourier transform-ion cyclotron MS [101], gas chromatography-mass spectrometry (GC-MS) [102], and nuclear magnetic resonance (NMR) [103].

**Figure 3:**
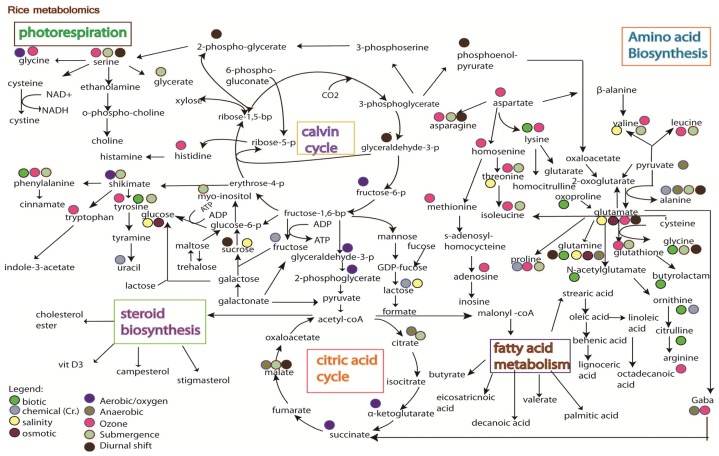
Metabolite changes in rice under biotic stress (bacteria) [109], chemical (Cr.) [110], ozone [68], anaerobic [111], aerobic [112], submergence [113] and metabolite changes under diurnal cycle [115].

**Figure 4:**
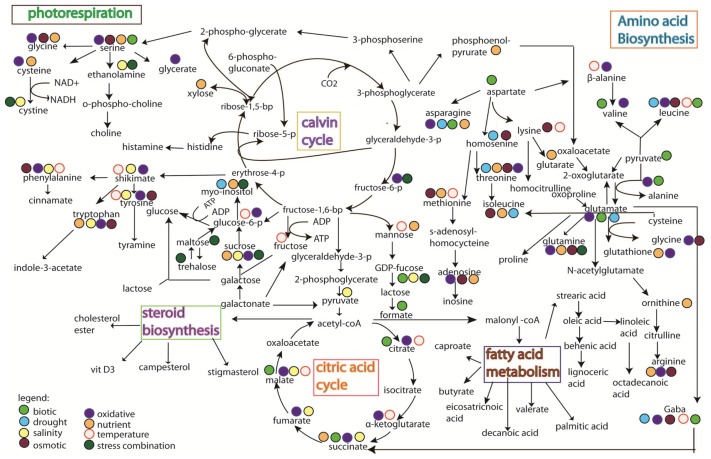
The changes of metabolites under different stress treatments. Results were obtained from treatment with biotic stress [118], drought [117], salinity [116], oxidative stress [121], temperature [102], osmotic stress [119], nutritional stress [101], and combination of stresses [122].

Studies on rice metabolomics have so far focused on the quality of metabolites, such as the types of metabolites that can promote seed germination [104], the metabolites variation between mutant and wild type plants [105], the profiling of metabolome at different developmental stages [106], and the observation of natural metabolite variation between rice varieties [107]. Limited information is available on the influence of environmental stresses on rice, except with a few studies reported on stress treatment with drought and salt [108], biotic (bacteria) [109], chemical (Cr.) [110], ozone [68], anaerobic [111], aerobic [112], submergence [113], oxidative [114] and metabolite changes under diurnal cycle [115] (Figure 3). Stress mechanisms have also been reported in other plant species, such as salt stress on tomato [100], nutrient deficiency that led to metabolites shifting in *Arabidopsis*[101,102,116], metabolite shift in *Arabidopsis* by temperature stress [102], drought stress on pea [117], biotic stress on opium poppy [118], and drought stress on *Thellungiella salsuginea*[119]. These studies have pinned down several key metabolism pathways that respond specifically to certain stresses (Figure 4).

In general, the integration pathways (Figures 3 and 4) show that abiotic and biotic stress pathways shifted the normal amino acid biosynthesis pathway, citric acid cycle, and photorespiration pathway. Glutamate, which acts as a stress biomarker, was observed in almost all the stress treatments. Threonine, glutamine, aspartate, isoleucine, and tyrosine were activated in both rice and other species studied (Figures 3 and 4), suggesting the involvement of these molecules in stress adaptation. The huge shifting observed in the amino acids biosynthesis pathway is likely due to the activation of their precursor in the central energy metabolism (observed in proteomics study). Changes in glycolytic activity could affect the availability of carbon for downstream response in amino acid synthesis [114], which may cause protein degradation. However, the changes of metabolites observed in Figures 3 and 4 are reports from various plant types, which may have different tolerance ability towards stress. Moreover, the protein degradation in plants is a complex process involving a multitude of proteolytic pathways. Thus, it remains to be investigated as to whether the accumulation of amino acids observed in all the stresses is a signal of protein degradation. It has also been proven that the accumulation of amino acids was correlated with the blocking of starch production under revert environmental challenge [120]. As starch is the main component for rice grain, reduction of starch content in rice plant could lead to huge losses in rice yield.

A completely different pattern of metabolomic response was observed when comparing the oxidative response in *Arabidopsis* with that in rice plant (Figures 3 and 4) and this is likely due to the differences in respect to oxidative stress. For example, submergence could cause less oxygen uptake and accumulation of toxin in plant cells, whereas the treatment of plant cells with methyl viologen (MV) could induce the accumulation of ROS. In addition, biotic stress could cause an oxidative burst to protect plant cells and ozone stress could contribute to the major phytotoxic air pollution. The integration maps (Figures 3 and 4) also indicates that ozone treatment in rice and MV treatment in *Arabidopsis* seem to induce similar responses. It was also revealed in an *Arabidopsis* study that 11 out of the 29 metabolites observed under oxidative response were induced by biotic stress. However, the mechanism as to how the biotic stress stimuli leads to oxidative damage remains to be investigated. Nevertheless, similar metabolic pathways (as discussed above) were also observed in rice suspension cultured with overexpressing Bax inhibitor-1 (BI-1) under oxidative stress [114]. Bax inhibitor-1 (BI-1) that functioned as the homologs of mammalian apoptotic machinery mediated the dynamic metabolic changes, including the depletion of the central metabolic pathway, redox imbalance, and accumulation of amino acids [114].

Some key metabolites, e.g., alanine, cysteine, cystine, glucose-6-phosphase, and GDP-fucose, showed obtuse response in rice plant compared to those in other species (Figures 3 and 4). The metabolites observed were found responding to redox activation of plastidic glucose-6-phosphase, which regulates the chloroplast enzyme [123]. Even though these metabolites are not detected in rice, photosynthesis-related proteins were still detected under ozone treatment [68]. Succinate in TCA cycle was also profoundly found under salinity, oxidative, and nutrient stress in *Arabidopsis*, whereas it was found present only under aerobic stress in rice (Figures 3 and 4). Succinate is well known as an oxidative damage indicator and the accumulation of citrate, observed at the beginning of the TCA cycle, implies the start of oxidative damage by H_2_O_2_. However, the toxicity of citrate accumulation is usually reduced or detoxified by α-ketoglutarate in order to reduce the accumulation of succinate [124]. In anaerobic and submergence stress (Figure 3), the recovery of oxidative stress seems to have occurred as no accumulation of succinate can be observed (as shown in Figure 3), yet future work (e.g., proteomic studies) is needed to corroborate this finding. On the contrary, glucose and proline were found regulated in rice plant but not in other plant species under stress conditions. Both glucose and proline are two metabolites that are well known as compatible osmolyte for osmotic adjustment under stress condition. Interestingly, proteomic result shows the regulation of glutamine synthetase [96] that functions in proline production [125].

The development of metabolomics data processing allows the identification of key metabolites that are specific to a stress pathway. However, it remains a difficulty to conclude the role of the specific pathway in certain stress conditions. This is due to the lack of a data profile to demonstrate whether the involved pathway is being up or downregulated. It is envisaged that future work could be performed to study the metabolic flux as a solution to the problem above; the work performed by Lehmann *et al.*[126], who conducted 13C redistribution analysis to prove the downregulation of glycolysis under oxidative stress treatment, could be a good example.

Another way to effectively identify oxidative stress biomarkers is through the use of stringent statistical validation to analyze the complexity of the multivariate data [127]. However, limited breakthrough is achieved in metabolomics due to several factors: (1) Massive replications of sample are needed for data verification due to the complex nature of the biological system that also changes diurnally. (2) Identification of hundreds of metabolites is difficult (especially in complex overlapping spectra) with limited search engines available to date. (3) It is a difficult task to integrate metabolomic data obtained from different analytical approaches, e.g., data from GC-MS and NMR provide different information, thus making the data integration more difficult. Since the integration study of the transcriptomic, proteomic, and metabolomics aspects are needed for the discovery of oxidative stress perception and signaling mechanism, an immediate effort is needed to develop a more powerful database that could provide metabolomics data obtained from different techniques and incorporate novel computational methods for “omic” integration study.

## 6. Summary of Omic’s Data Set and the Challenge of Integrating Multi-Omic Data Sets

The results from proteomic studies showed huge overlapping in central metabolism (e.g., Calvin cycle, carbohydrate, and lipid biosynthesis pathway) under drought, salinity, and heat treatment (Table 2). However, different metabolism pathways were found to regulate and adjust under metabolomic level, particularly the photorespiration, amino acid biosynthesis, and citric acid pathway. This is likely due to the involvement of PTM and downstream enzymatic response instead of cellular damage responses, which induce more complex metabolites pathways. This indicates the weakness in the use of proteomic approach (as discussed above)—the proteomic approach assumes that the increase of abundance in protein level is always accompanied by biological active compound, but in reality it may encompass the factors by posttranslational modification, which may modify the activities and characteristics of the proteins. An example to support this is the poor correlation between the protein expression level and the microarray results observed in short-term stress treatment [128,129].

The dynamics of antioxidant response has been observed in the proteomic study; however, the link to its downstream responses is missing. Therefore, the role of the metabolite shifting observed at the metabolomic level is unclear, e.g., are those changes related to the regulation of stress perception, or are the changes just the signal of indicating plant damage as a result of lethal stress? The research of Urbanczyk-Wochniak *et al.*[130] showed that only 571 out of 26,616 of pair-wise transcript–metabolite correlation analyses showed significant correlation. Therefore it can be concluded that the development of bioinformatics in connecting the response in transcription level to either proteomic or metabolomic changes is yet to be completed.

The rapid progress in omics research has led to more and more data sets being generated across all branches of life sciences studies. Various analytical applications, which are essential for the effective integration of data resources, have been reported in different databases [131,132]. These massive datasets are obtained via four important stages: (i) data generation that involves the generation of raw data, e.g., microarray experiment and DNA chips; (ii) data processing that involves the pre-processing of raw data through the use of a computer method, e.g., baseline correction and cutting out noise spectra in NMR experiment; (iii) data integration that arranges data into a biological meaningful way; and, as the last step, the (iv) data analysis that integrates omics data into a functioning systemic context as a whole [133]. However, recent progress in bioinformatics has indicated that the majority of multi-omic researches are still in the first three stages due to the lack of resources allocated for data integration study [133]. This, however, raises the issue on the validation of single annotations in a recent study because the reliability of the data sets should be integrated and tested in order to avoid interpretation of false–positive and false–negative results [134]. The challenge in the integration of omic data analysis has been discussed [135]. It was reported that the major problem arose from the incomplete and diverse form of information available on bioinformatics across many sources of data. Therefore, algorithmic approaches have been proposed as the solution for these problems [136].

Integrative omics–metabolic analysis (IOMA) has been built to integrate proteomic and metabolomic data with genome-scale metabolic models in order to accurately predict the cellular metabolism [137]. Recently, a user-friendly web server, SteinerNet, has been created that enables the integration of high-throughput data from different cellular condition and it has been reported to be able to present the results in a biologically meaningful pathway [138]. However, it was revealed that no data on plants has yet been analyzed by either IOMA or SteinerNet. Nevertheless, the use of graph-based integration method (Ondex) has shown its strength as an alternative method in presenting metabolic pathways and protein–protein integration data in *Arabidopsis*[139]. There is also a method developed for multidimensional omics data processing called OmicBrowse, which is suitable for plant breeding purposes [140].

It was revealed that the transfer of biomarkers for a crop improvement program is still a process filled with many pitfalls and limitations, and it is mostly limited by structural and scientific factors. Before the application of a potential biomarker for use in agronomy, the potential biomarker should be confirmed and validated using hundreds of specimens and it should also be specific, sensitive, and reproducible. The major problem in the discovery of biomarkers is the low quality of biomarker validation, which could be attributed to:

(1) Weak biomarker characterization and validation strategies;
(2) Limitation of the analytical techniques used. As shown in Figures 3 and 4, it is hard to conclude whether the differences in metabolites identified from the same stress is due to species-specific response, or instead merely reflects the variation between analytical tools, e.g., the use of GC *versus* the use of NMR;
(3) Difficulty in obtaining highly sensitive and specific biomarkers.

Metabolites may shift in massive amounts even with slight changes of environmental condition. Therefore, new research in biomarkers needs to be performed in order to address the accuracy, precision, and reliability in measuring relevant physiological signs, and to avoid the choosing of false–positive results.

## 7. Conclusions

The stress-adaptation mechanism is a complex system that involves the reaction of several enzymes and also metabolites with multiple overlappings in different cell compartments due to possible discrepancies in tolerance levels. This review presents and discusses mainly the key findings in the development of “omic” technology in rice systems. Unfortunately, despite the marvelous progress shown in bioinformatics, the integration of data outputs from phenome, transcriptome, proteome, and metabolome (e.g., the detection of PTM proteins and metabolic changes) has been found inefficient as of yet. Hence, the recent progress in omic technology shows the discovery of only a part of the adaptation system and is yet to be sufficient to tell the whole story of stress functioning and signaling mechanism in plants. Therefore, more resources should be allocated for development of bioinformatics technologies in order to effectively target the right biomarkers for crop improvement.

## Figures and Tables

**Table 1:** ROS-scavenging enzymatic antioxidants and their roles in transgenic rice for abiotic stress tolerance.

Gene	Source	Response	Reference
**Superoxide dismutase (SOD)**			

Cu/Zn SOD	*Avicennia marina*	Transgenic plants were more tolerant to methyl viologen (MV)-mediated oxidative stress, salinity, and drought stress.	[49]
Mn SOD	*Pisum sativum*	Electrolyte content declined and less injury observed in leaf discs of transgenic plants compared to that observed in wild type plants following treatment with polyethylene glycol (PEG).	[50]
Mn SOD	Yeast	Transgenic plants maintained high level of SOD and ascorbate peroxidise activity in chloroplast upon exposure to salt stress, while decrease in SOD activities was observed in wild type plants.	[51]

**Catalase (CAT)**			

CAT KatE	*E.coli*	Transgenic rice plants showed at least 1.5–2.5 fold increase of CAT upon exposure to salt stress. The transgenic rice can survive for another 14 days compared to wild type plants following treatment with salt stress.	[52]
CAT	*Triticum aestivum* L.	In 5 °C of cold stress, CAT was found 4–15 times higher in transgenic plants compared to that observed in wild type plants with very low levels of H_2_O_2_.	[53]
CAT	*Suaeda salsa*	Co-expression of CAT and GST resulted in the increment of SOD and CAT activities following treatment with salt and paraquat stress, while GST activity only increased upon treatment with paraquat stress. H_2_O_2_, malondialdehyde, and electrolyte leakage were found to decrease in transgenic rice compared to wild type plants.	[54]

**Ascorbate peroxidase**			

Knockdown *OsAPx1* and *OsAPx2*	Rice	Compensation of ascorbate peroxidase by other peroxidases, including glutathione peroxidase (GPX).	[55]
OsAPXa	Rice	Transgenic plants maintained H_2_O_2_ and malondialdehyde (MDA) content when subjected to cold stress.	[56]
Silencing APx1/2s	Rice	Upregulation of other peroxidases was observed under salinity, heat, high light, and MV treatment.	[55]

**Glutathione reductase**			

GR	*Brassica campestris*	High expression of GR improved the protection against photo-bleaching of chlorophyll and photo-oxidative action of MV in thylakoid membranes at 25 °C.	[57]

**Glutathione*S*-transferases**			

GST	Rice	Seedlings of the transgenic lines grown under submergence demonstrated enhanced germination and growth rates at low temperature.	[58]
OsGSTL1	Rice	The overexpression lines showed an increase in GST and GPX activities, and a decrease in the level of superoxide was observed.	[59]
GST	*Suaeda salsa*	Salt and paraquat stress tolerance were observed due to GST, CAT, and SOD activity.	[54]

**Table 2:** The change of protein profile stimulated by diversity of abiotic stress.

Genotype	Treatment	Major result	Reference
*Oryza sativa* L. cv CT9993 and cv IR62266	Drought	Signal transduction:Translation elongation factor, actin depolymerizing factor Energy metabolism: ribulose-1, 5-bisphosphate carboxylase/oxygenase (RuBisCo) activase, Triosephosphate isomerase Antioxidant: superoxide dismutase (SOD), GSH-dependent dehydroascorbate reductase, Unknown function: *S*-like RNase homolog, isoflavone reductase-like protein	[77]
*Oryza sativa L.* Nipponbare Zhonghua 8	2–6 days drought	Defense:-superoxide dismutase (SOD), salt-induced protein (SALT), Energy metabolism: chloroplast ATPase, RuBisCO small subunit, RuBisCO large subunit, photosystem II oxygen-evolving complex protein, oxygen-evolving enhancer protein, light harvesting complex chain II Cell structure: Cys peroxiredoxin BAS 1 Signal transduction: actin depolymerizing factor Unknown function: serine hydroxymethyltrasferase I, phosphoglucomutase cytoplasmic	[86]
*Oryza sativa L.* Nipponbare, IR36, Pokkali	Salinity 50 mM NaCl, 24 h	Energy metabolism: photosystem II oxygen-evolving complex protein, oxygen evolving enhancer protein (OEE 2), fructosebisphosphate aldolases Antioxidant-superoxide dismutase (SOD)	[87]
*Oryza sativa L.* IR 4630-22-2- 5-1-3	Salinity 50 mM NaCl, 7 days	RuBisCo activase (RCA): RuBisCO activase Iron homeostasis: Ferritin, Energy and metabolism: ATP synthase-putative phosphoglycerate kinase, Antioxidant: SOD Metabolism synthesis: *S*-adenosyl-l-methionine synthetase. Cell cycle: Translation initiation factor 5A	[88]
*Oryza sativa L.* cv. Nipponbare	Salinity 150 mM Nacl, 24 h, 48 h and 72 h	Glycolysis enzyme: Triosephosphate isomerase, Enolase Signal transduction: UDP-glucose pyrophosphorylase (UGPase) Energy generation: Cytochrome c oxidase subunit 6b-1 (COX6b-1), nascent polypeptide-associated complex alpha chain, *S*-adenosylmethionine synthetase 2 Antioxidants: Glutamate synthetase, Peroxidase Unknown function: Putative actin-binding protein and putative splicing factor-like protein	[75]
*Oryza sativa L.* cv. Nipponbare and Zhonghua 8	Osmotic Mannitol 400 mM, 48 h	Redox homeostasis: glutathione S-transferase (GST) Heat shock proteins: heat shock protein, dnaK-type molecular chaperone, endosperm luminal binding protein (BiP), Housekeeping: 26S proteasome regulatory subunit, Signal transduction: calreticulin precursor Lipid accumulation: lipid transfer protein, Glyoxalase–glyoxalase I Proteasome regulatory pathways: 20S proteasome α-subunit, proteasome-degradation system-related proteins, endoplasmic reticulum (ER)-related proteins Cell death-related protein: uroporphyrinogen decarboxylase	[89]
*Oryza sativa L*.	Osmotic 20% PEG, 8 days	Redox metabolism; Prx and putative thioredoxin peroxidase Photosynthesis-rbcS and rbcL Cytoskeleton stability: putative actin-binding protein, ABP Defense: putative chitinase Protein metabolism: ribonuclease Signal transduction: voltage-dependent anion selective channel protein and osmotin-like protein	[90]
*Oryza sativa L.* cv. Dongjin	Heat 42 °C, for 12 and 24 h	Heat shock proteins: HSP 70, dnak-type molecular chaperone, endosperm luminal binding protein (Bip), putative chaperonin 60 (Cpn 60) precusor Energy and metabolism: related protein-Transketolase, UDPglucose pyrophosphorylase, putative thiamine, pyruvate dehydrogenase complex (PDC) Redox homeostasis: GST, dehydro-ascorbate reductase (DHAR), thioredoxin h-type, SOD Regulatory proteins/ housekeeping enzymes: chloroplast elongation factors, cysteine proteinase, proteosome subunit alpha type1 and subunit of 20s proteosome, nucleoside diphosphate kinase 1 (NDPK1)	[91]
*Oryza sativa L.* ssp. *japonica*	Cold 15, 10 and 5 °C 24 h	Signal transduction: Elongation factor Metabolism synthesis: *S*-adenosylmethionine synthetase 2, VB12-independent methionine synthase Antioxidative: GDP-mannose 3′,5′-epimerase Protein metabolism: chaperonin, ATP-dependent Clp protease ATP-binding subunit Oxygen-evolving complex proteins: NADH-ubiquinone oxidoreductase, putative ferredoxin-NADP(H) oxidoreductase	[92]
*Oryza sativa L.* cv. Nipponbare	Cold 5 °C 48 h	Cellulose synthesis: UDP-glucose pyrophosphorylase Energy metabolism: adenylate kinase protein, RuBisCO LSU, vacuolar ATPase B subunit, H^+^ transporting ATP synthase, fructose-bisphosphate aldolase Protease: cysteine proteinase, 5-methyltetrahyropteroyltriglutamate-homocysteine *S*-methyl transferase, protein disulfide isomerase, Stress defense: Betaine aldehyde dehydrogenase (salt), Phenylalanine ammonia lyase (mechanical wounding), Beta-1,3-glucanases Signal transduction: Calreticulin, phosphoglycerate kinase, Elongation factor G Heat shock protein: HSP70 Housekeeping enzymes: nucleoside diphosphate kinase (NDPK) Antioxidant enzymes: superoxide dismutase (Cu/Zn), catalase, Unknown function: phosphoglucomutase, chitinase III-like protein, malate dehydrogenase	[93]
*Oryza sativa L.* cv. Dongjin	Cold 5 °C 12 h, 24 h, 36 h 10 °C 24 h and 72 h	Antioxidant enzymes: Ascorbate peroxidase, putative glutathione *S*-transferase, thioredoxin *h*-type (Thx *h*) and thioredoxin peroxidase Housekeeping enzymes: nucleoside diphosphate kinase 1 (NDPK1) Lipid-binding protein-fibrillin-like protein Protease: cysteine proteinase Regulatory: drought-inducible late embryogenesis abundant protein, RING zinc finger protein-like	[94]
*Oryza sativa L.* Chunyou 58 and Yongyou 6	Nitrogen Shortage of N for 12 h, 3 days and 7 days	Photosynthetic metabolism: ribulose-1,5-bisphosphate carboxylase/oxygenase activase, type II tight-harvesting chlorophyll a/b-binding protein, carbonic anhydrases, rubisco large subunit, 23kDa polypeptide of photosystem II, dTDP-glucose 4–6-dehydratase-like protein and H protein subunit of glycine decarboxylase 3′-partial Stress responses/defenses: DegP2, harpin-binding proteins, heat shock-related proteins, glutathione *S*-transferase GSTF14, Fibrillin-like protein, Glyceraldehyde-3-phosphate dehydrogenase Membrane transporter: Putative chloroplast inner envelop protein, SecA protein	[95]
*Oryza sativa L.* cv. Dongjin	Chemical treatment 100 μM CdCl_2_ for 24 h	Antioxidant enzymes: l-ascorbate peroxidase 1, GR, glutathione *S*-transferases, NADH-ubiquinone oxidoreductase, hypothetical protein, peroxidase, putative ferredoxin-NADP(H) oxidoreductase Carbohydrate metabolism: Bisphosphoglycerate-independent phosphoglycerate mutase, glyceraldehyde-3-phosphate dehydrogenase, Alpha-1,4-glucan-protein synthase, endo-1,3-betaglucanase Amino acids and photosynthesis metabolism: glutamine synthetase, Photosystem II oxygen-evolving complex protein 2, ribulose bisphosphate carboxylase/oxygenase activase Protein metabolism: Putative ubiquitin isopeptidase T, 26S proteasome, Chloroplast translational elongation factor Tu, elongation factor P, Putative chaperonin 60 beta, vacuolar proton-ATPase, guanine nucleotide-binding protein subunit beta-like protein, ricin B-related lectin domain-containing protein	[96]
*Oryza sativa L.* cv. Nipponbare	Ozone 0.2 ppm, 24 h	Cellular processing and signaling: Ion transporters, MAPK, Ca^2+^-dependent protein kinase (CPKs), Ca^2+^-binding proteins, receptor kinases Photosynthesis: ATP-dependent Clp protease, chloroplast cell division protease ftsH homologous, HSP 90, Rubisco Defense: chloroplast L-APX, glutathione peroxidase, putative basic secretory protein Antioxidant: glutathione *S*-transferase, glutathione peroxidase, glutathione reductase, catalase, monodehydroacorbate reductase	[68]
